# Predictive value of the neutrophil-to-lymphocyte ratio in the prognosis and risk of death for adult sepsis patients: a meta-analysis

**DOI:** 10.3389/fimmu.2024.1336456

**Published:** 2024-03-18

**Authors:** Hongsheng Wu, Tiansheng Cao, Tengfei Ji, Yumei Luo, Jianbin Huang, Keqiang Ma

**Affiliations:** Department of Hepatobiliary Pancreatic Surgery, Huadu District People's Hospital of Guangzhou, Guangzhou, Guangdong, China

**Keywords:** adult sepsis, NLR, prognosis, meta-analysis, mortality

## Abstract

**Background:**

The neutrophil-to-lymphocyte ratio (NLR) is a commonly used biomarker for acute inflammation that often rises during sepsis, making it a valuable diagnostic indicator for clinical practice. However, no consensus has been reached on the prognostic value of NLR for predicting the prognosis and mortality risk in adult sepsis patients. In light of this controversy, we conducted a meta-analysis to clarify the prognostic significance of NLR in adult sepsis patients. The meta-analysis was registered in the PROSPERO database (registration number CRD42023433143).

**Methods:**

We performed a comprehensive literature search in PubMed, Cochrane Library, Ovid, and Springer databases, using retrieval terms “*sepsis*” or “*septic shock*” and “*prognosis*” or “*mortality*” for studies published between January 1, 2000, and May 31, 2023. Children and neonates with sepsis were excluded from our research. Two independent researchers conducted the literature search and data extraction. Consensus was reached when discrepancies occurred, and in case of persistent discrepancies, the final decision was made by the research supervisor. The hazard ratio (HR) and its corresponding 95% confidence interval (95% CI) were extracted from each study included in the analysis. A random-effects model was used to synthesize all HRs and their 95% CIs. Sensitivity analysis was performed to investigate heterogeneity. Sensitivity analysis was conducted to identify studies that had a significant impact on the overall results of the meta-analysis. Subgroup analysis and meta-regression were performed to explore sources of heterogeneity. Egger’s test was also used to investigate publication bias in this meta-analysis.

**Results:**

After a comprehensive literature search and screening, we included 12 studies comprising 10,811 patients for the meta-analysis. The pooled results indicated that patients with a higher NLR level were associated with a poor prognosis (Random-effects model, HR: 1.6273, 95% CI: 1.3951-1.8981). Heterogeneity testing showed significant heterogeneity (I^2 =^ 87.2%, 95% CI: 79.5-92, p<0.0001). Sensitivity analysis was performed to investigate the sources of heterogeneity, which revealed that the omission of one highly sensitive study significantly reduced the I^2^ value. After removing this study, a strong association was found between a higher NLR level and poor prognosis and risk of death in adult sepsis patients (Random-effects model, HR: 1.6884, 95% CI: 1.4338-1.9882). Both subgroup analysis and meta-regression indicated that the study design and testing time of NLR were sources of heterogeneity. Egger’s test showed no obvious publication bias in this meta-analysis.

**Conclusion:**

NLR is a reliable and valuable biomarker for predicting prognosis and the risk of death in adult sepsis patients.

**Systematic Review Registration:**

[https://www.crd.york.ac.uk/prospero/display_record.php?ID=CRD42023433143] PROSPERO, identifier [CRD42023433143].

## 1 Introduction

Sepsis is an infectious disease characterized by high mortality and poor prognosis (1, 2). In 2016, The Third International Consensus Definitions for Sepsis and Septic Shock redefined sepsis as an infection accompanied by organ dysfunction (3, 4). Over the years, the Sequential Organ Failure Assessment (SOFA) score has been increasingly adopted to diagnose and assess the prognosis of sepsis (5, 6). While the SOFA score is a valuable tool for sepsis evaluation, it involves numerous parameters and indexes, which can be cumbersome during the assessment process. Accordingly, significant efforts have been undertaken to explore new diagnostic techniques and prognostic biomarkers (7, 8).

One such biomarker is the neutrophil-to-lymphocyte ratio (NLR), which is derived from the ratio of neutrophil count to lymphocyte count and can be obtained from a complete blood count test. It has been established that elevated NLR levels indicate acute infectious inflammation and are commonly considered an inflammatory biomarker (9, 10). There is an increasing consensus suggesting that NLR plays a crucial role in predicting sepsis and can be a valuable marker for sepsis diagnosis (11–13). Besides, a high NLR level has been associated with poor outcomes in adult sepsis patients. However, a recent prospective study by Schupp, T et al. (14) contradicted these findings, stating that NLR was not a reliable parameter to differentiate between patients with sepsis and septic shock, nor could it predict prognosis. Consequently, the utility of NLR as a biomarker for sepsis prediction remains uncertain. To bridge this knowledge gap, we conducted a comprehensive search and analysis of available studies to investigate the value of the neutrophil-to-lymphocyte ratio in predicting prognosis for adult sepsis patients.

## 2 Materials and methods

### 2.1 Literature search strategy

We conducted a comprehensive search for literature on adult patients diagnosed with sepsis or septic shock between January 1, 2000, and May 31, 2023, from the Pubmed, Cochrane Library, Ovid, and Springer databases using the following MeSH search headings: “*sepsis*” or “*sepsis shock*” and” *neutrophil-to-lymphocyte* ratio” or “*NLR*” and “*prognosis*” or “*mortalit*y”. To expand the search scope, we also utilized the “related articles” function of Pubmed and searched the references of identified articles simultaneously. The retrieved studies included prospective and retrospective study designs.

### 2.2 Inclusion and exclusion criteria

The inclusion criteria for this meta-analysis were as follows: (1) Studies involving hospitalized adult patients with sepsis or septic shock; (2) Definition of sepsis or septic shock and corresponding management according to international guidelines (3, 18); (3) Use of NLR as an evaluating indicator for predicting sepsis or septic shock; (4) Availability of Hazard Ratio (HR) and the corresponding 95% Confidence Interval (95% CI) that could be extracted from the study with Cox model or Kaplan-Meier stratification validation. The exclusion criteria were as follows: (1) Studies involving neonatal or pediatric sepsis; (2) Studies involving animal experiments, systematic reviews, case reports, or letters; (3) Studies providing only odds ratio (OR) from univariate analysis or logistic regression; (4) Studies from which data could not be extracted. The process of inclusion and exclusion criteria for this meta-analysis strictly followed the PRISMA 2000 procedure.

### 2.3 Data extraction and quality assessment

Data extraction from each included study involved retrieving the name of the first author, publication year, region of the population, study design, total number of patients, number of survival and non-survival patients, time for mortality observation, time of NLR testing, cut-off NLR value, HR, and the 95% CI for prognostic prediction. Two independent reviewers performed data extraction, and disagreements were resolved through discussion and consensus. If further disagreements occurred, they were resolved by senior authors (KQ Ma and H.S*. Wu*). Quality assessment of the included studies was performed using the Newcastle-Ottawa Scale (NOS), evaluating the exposed cohort, comparability, outcome of interest, assessment of outcome, and cohort follow-up (15). The maximum score in this assessment system is 9 points, with literature with a score of no less than 6 points considered high quality.

### 2.4 Statistical method

For the preliminary synthesis of HRs and their corresponding 95% CIs, the “*metagen*” function from the “*meta*” package was used. Heterogeneity was tested using I^2^, where I^2^ <50% indicated nonsignificant heterogeneity and a fixed-effects model was used for data synthesis. Conversely, when the I^2^ value was ≥50%, a random-effects model was used. Sensitivity analysis was conducted to investigate highly sensitive literature with the “*metainf*” function, and data re-synthesis was performed after eliminating highly sensitive literature. Subgroup analysis with the “byvar” function and meta-regression with the “metareg” function were used to explore sources of heterogeneity. Publication bias was investigated using an enhanced contour funnel plot and Egger’s test. All statistical analyses and figure generation were performed using R software Version 4.1.3.

## 3 Results

### 3.1 Identification of relevant studies

**Figure 1:**
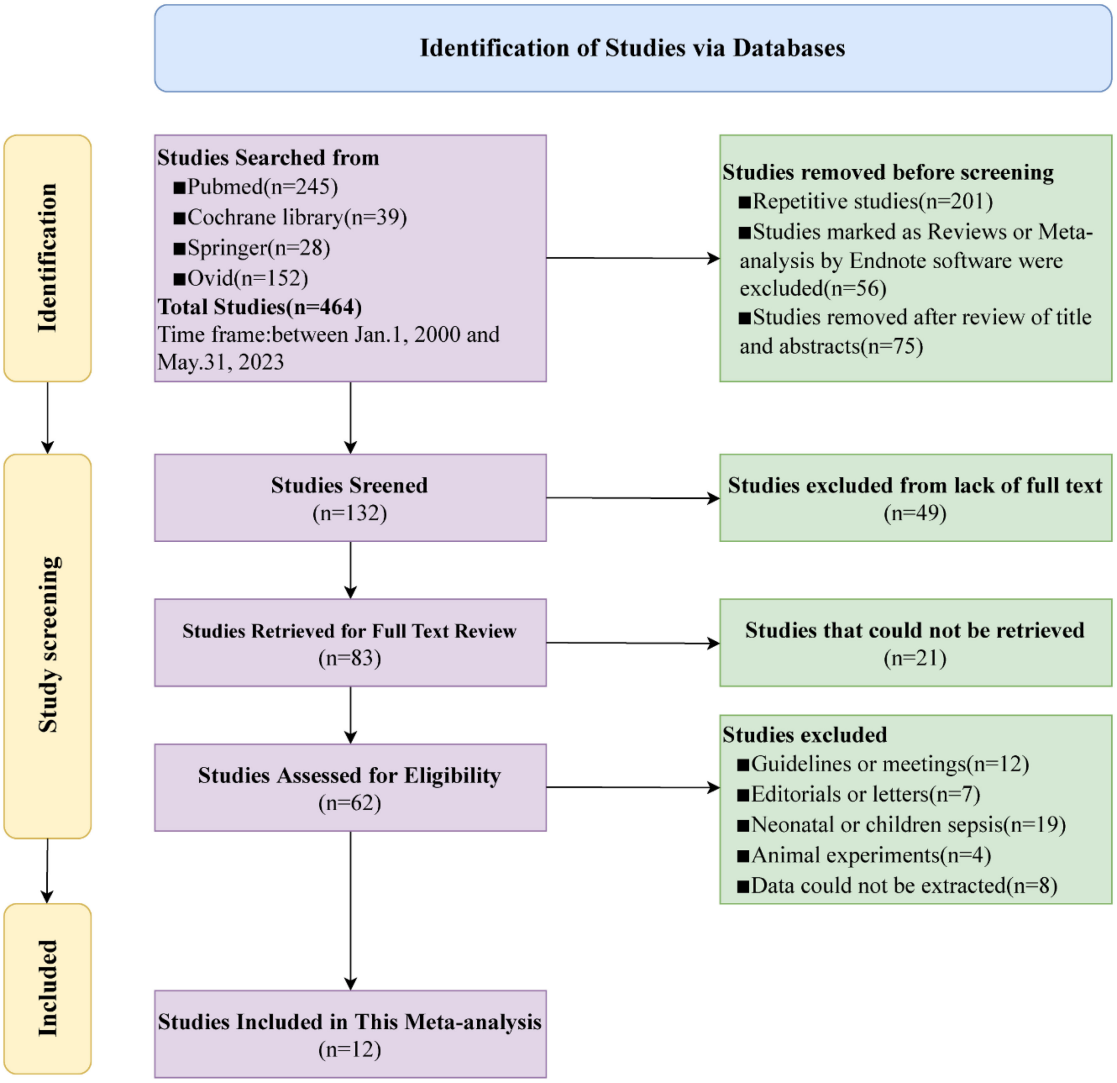
Flowchart for selection of studies included in this meta-analysis based on PRISMA guidelines.

464 relevant studies were initially identified from the Pubmed, Cochrane Library, Springer, and Ovid databases. After excluding duplications and ineligible studies, 132 studies remained. Further screening resulted in the exclusion of 49 studies due to the unavailability of the full text. Additionally, 21 studies were removed as they could not be retrieved, leaving 62 studies for eligibility. Finally, reviews (n=12), letters to the editor (n=7), studies about neonatal or children sepsis (n=19), animal experiments (n=4), and unavailable studies (n=8) were excluded. Ultimately, 12 studies with 10,811 patients were included in this meta-analysis (16–27). Among the included patients were 8,389 survivors and 2,422 deaths, with a non-survivor-to-survivor ratio of approximately 1:3.5. The flowchart of study selection and screening is depicted in **Figure 1**.

### 3.2 Study characteristics and quality assessment

Among twelve included studies, ten studies (16, 17, 19–21, 23–27) defined as sepsis, one defined as sepsis shock (22) and one defined as sepsis or sepsis shock (18). Three studies diagnosed sepsis based on the sepsis-2 definition (16–18) and other nine studies base on sepsis-3 definition (19–27). For the study design, three studies (17, 19, 26) were prospective and nine studies (16, 18, 20–25, 27) were retrospective. The mean age of patients in six studies (16–18, 20, 22, 25) was older than 60 years, while the remaining six studies involved patients younger than or equal to 60 years (19, 21, 23, 24, 26, 27). The observed outcomes varied among studies: seven studies (18, 20–25) used 28-day mortality as the observed outcome, two studies assessed 30-day mortality (19, 27), another two studies reported in-hospital mortality (17, 26), only one study (16) used 15-day mortality as the observed outcome. NLR was used as a prognostic risk factor for predicting adult sepsis in all 12 studies, and the cut-off values for NLR varied among studies. Six studies (17–20, 26, 27) defined their cut-off values as more than 10, while another six studies (16, 21–25) as less or equal to 10. The testing time for NLR also differed: seven studies (17, 19–21, 24, 26, 27) tested the NLR on day 1 of hospitalization, two studies (16, 18) on day 2, one study (22) on day 3 and the remaining two studies (23, 25) on day 7. The quality assessment using the Newcastle-Ottawa Scale (15) indicated that all included studies had a total score of more than 6 points, suggesting higher quality and a lower risk of bias. The characteristics and quality assessment of the included studies are presented in **Table 1**.

**Table 1:** Study baseline characteristics and quality assessment.

Author	Year	Country	Disease severity	Study design	Male/ Female	Mean age(yrs)	Patients NO.	Survival/Death	Outcome Measure	Time of sampling	Cut-off value	Comorbidities	Quanlity score
Terradas,R (16)	2012	Spain	Sepsis	Retrospective	1316/995	67.7	2311	2056/255	15-day mortality	Day2	7.00	NA	8
Akilli,NB (17)	2014	Turkey	Sepsis	Prospective	203/170	74	373	209/164	In hospital mortality	Day1	11.90	Coronary artery disease, Congestive heart failure, Chronic renal failure, COPD, Malignancy	9
Hwang,S.Y (18)	2017	Republic of Korea	Sepsis, Sepsis shock	Retrospective	787/821	65	1608	1397/211	28-day mortality	Day2	31.00	Hypertension, Diabetes, Cardiovascular disease, Chronic lung disease, Chronic renal disease, Liver cirrhosis	8
Lorente,L (19)	2020	Spain	Sepsis	Prospective	140/63	60	203	135/68	30-day mortality	Day1	12.10	Chronic renal disease, COPD, Diabetes, Ischemic heart disease	7
Weiyan,Y (20)	2020	China	Sepsis	Retrospective	1539/1504	67	3043	2433/610	28-day mortality	Day1	20.25	Atrial fibrillation, Coronary heart disease, Congestive heart failure, Diabetes, Malignancy, Chronic renal disease, Liver cirrhosis	8
Li,J.Y (21)	2021	China	Sepsis	Retrospective	168/106	57.68	274	79/195	28-day mortality	Day1	5.51	Hypertension, Coronary heart disease, Diabetes, COPD, Cerebrovascular disease, Chronic renal disease, Malignancy	8
Li,Q (22)	2021	China	Sepsis shock	Retrospective	840/405	69.56	1245	809/436	28-day mortality	Day3	6.56	COPD, Coronary heart disease, Heart failure, Hypertension, Diabetes, Liver cirrhosis, Maglinancy	7
Liu,Shuangqing (23)	2021	China	Sepsis	Retrospective	116/100	54.7	216	144/72	28-day mortality	Day7	4.18	Hypertension, Ischemic heart disease, COPD, Autoimmune disease, Chronic kidney disease	6
Liu,S (24)	2021	China	Sepsis	Retrospective	167/97	52.92	264	186/78	28-day mortality	Day1	4.94	Hypertension, Diabetes, Coronary heart disease, COPD	8
Liu,Y (25)	2021	China	Sepsis	Retrospective	60/31	65	91	71/20	28-day mortality	Day7	8.49	Hypertension, Coronary heart disease, Chronic renal failure, Diabetes, COPD, Malignancy	8
Chebl,R.B (26)	2022	America	Sepsis	Prospective	509/365	53	874	675/199	In hospital mortality	Day1	14.20	Chronic kidney disease, Hypertension, Dyslipidemia, Atrial fibrillation, Coronary artery disease, Congestive heart failure, Malignancy	7
Wei,W (27)	2023	China	Sepsis	Retrospective	217/92	57.8	309	175/134	30-day mortality	Day1	13.16	Hypertension, Diabetes, Coronary heart disease, Chronic kidney disease, Pneumonia, Acute respiratory distress syndrome, Malignancy	6

"NA" indicated that Comorbidities were no mentioned in the study.

### 3.3 Preliminary studies synthesis

**Figure 2:**
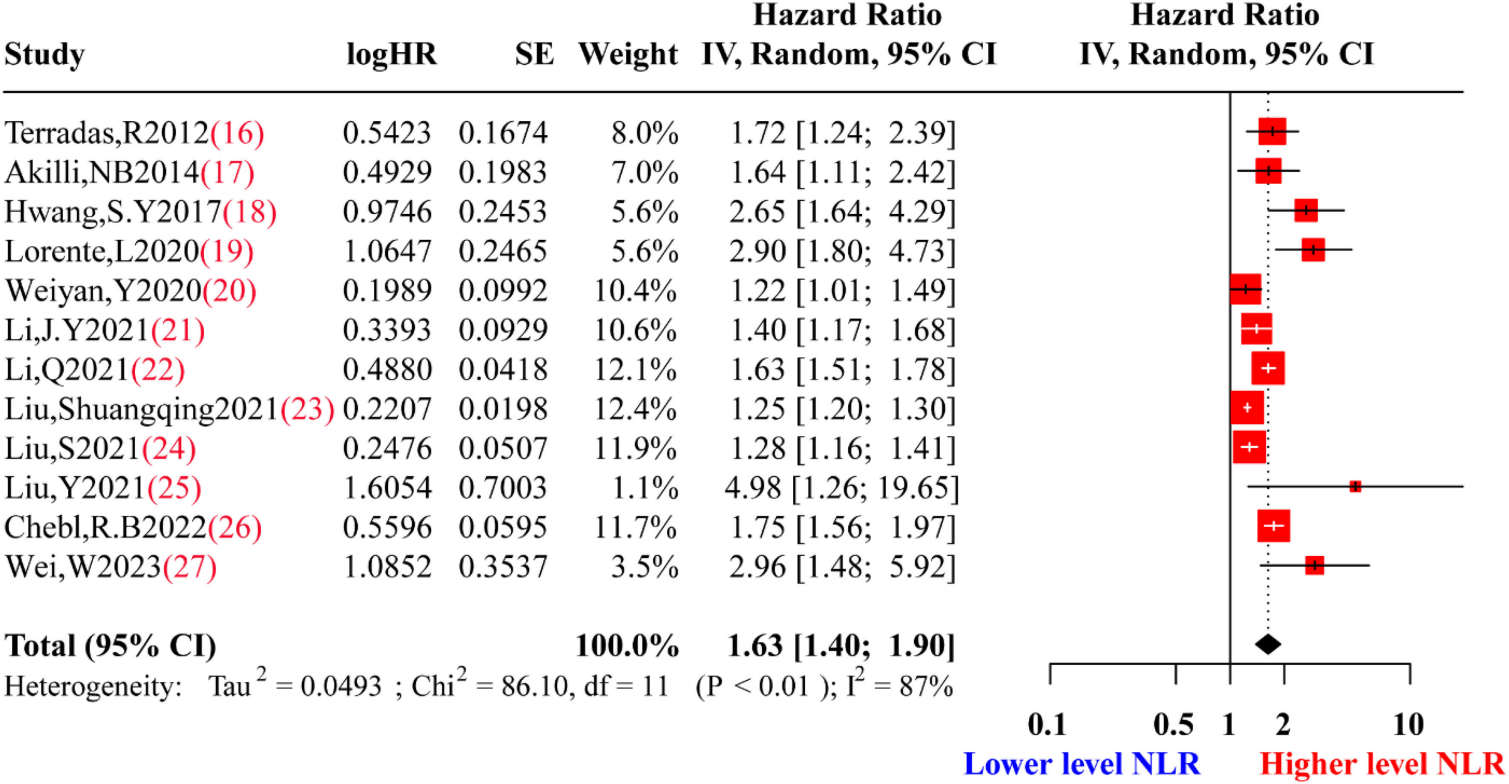
Forest plot of preliminary studies synthesis. HR, Hazard Ratio; SE, Standard Error; NLR, neutrophil-to-lymphocyte ratio. Red squares represent the point estimates of the HR of each study, with 95% CI indicated by horizontal bars. Black diamond represent the summary estimate from the pooled studies with 95%CI.

The pooled analysis of HRs and their corresponding 95% CIs extracted from the included studies indicated that higher NLR was associated with a poorer prognosis for adult sepsis patients (HR: 1.6273, 95% CI: 1.3951-1.8981) with significant heterogeneity (I^2 =^ 87.2%, p<0.0001), using a random-effects model (**Figure 2**).

### 3.4 Sensitivity analysis

**Figure 3:**
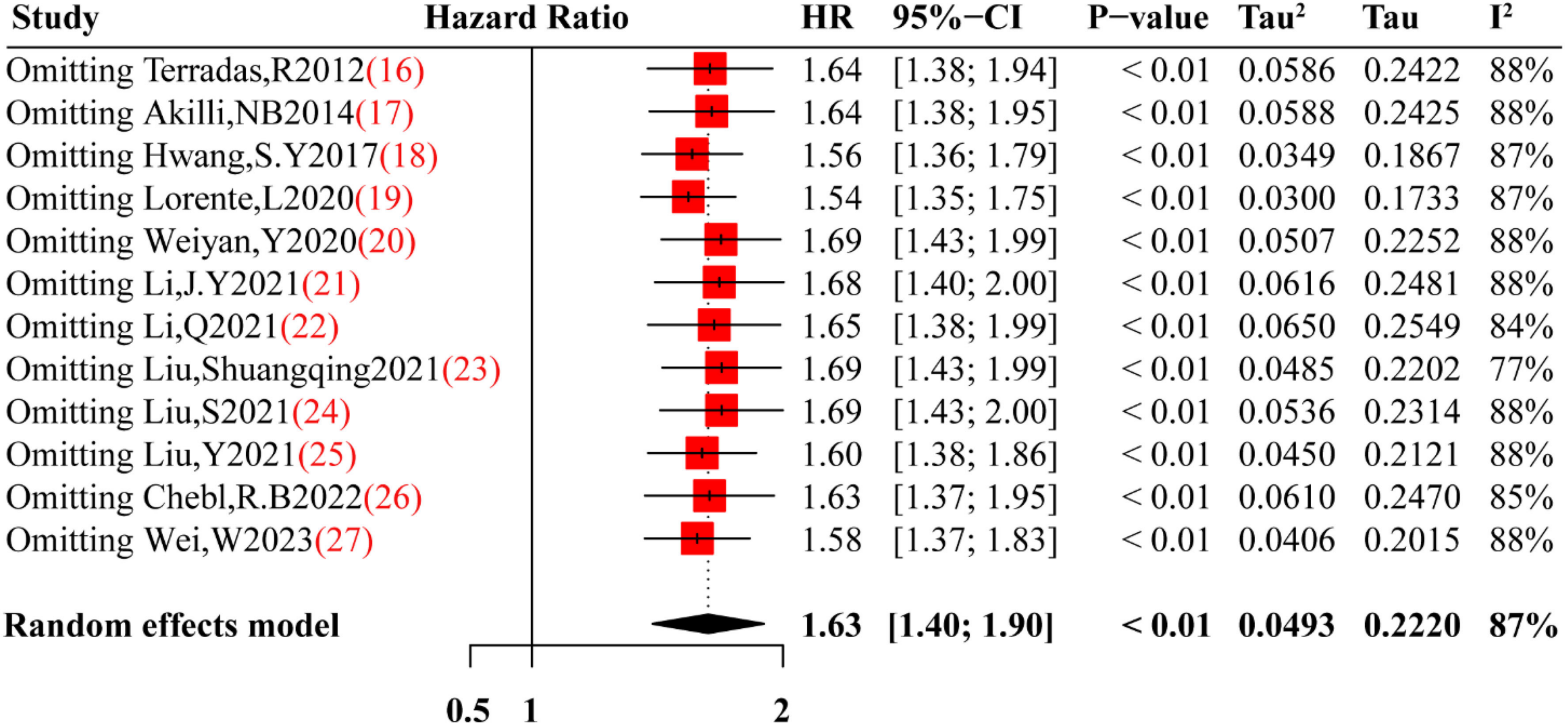
Sensitivity analysis of this meta-analysis. Red square are the point estimates of the omitting HR, with 95% CI indicated by horizontal bars. While black diamond is the heterogeneity from the pooled studies with 95% CI base on random effects model.

The purpose of sensitivity analysis was to examine the stability of results under certain hypothetical conditions and preliminarily investigate sources of heterogeneity in the included literature. High sensitivity literature meant that after excluding this literature, the heterogeneity of the meta-synthesis had a significant decrease. Given the significant heterogeneity observed in the preliminary synthesis, sensitivity analysis was performed to identify potentially highly sensitive literature. One study by Liu, Shuangqing et al. (23) contributed significantly to the high level of heterogeneity, and its exclusion reduced the I^2^ to 77% (**Figure 3**).

### 3.5 Re-synthesis of the included studies and correlation of the NLR to disease severity

**Figure 4:**
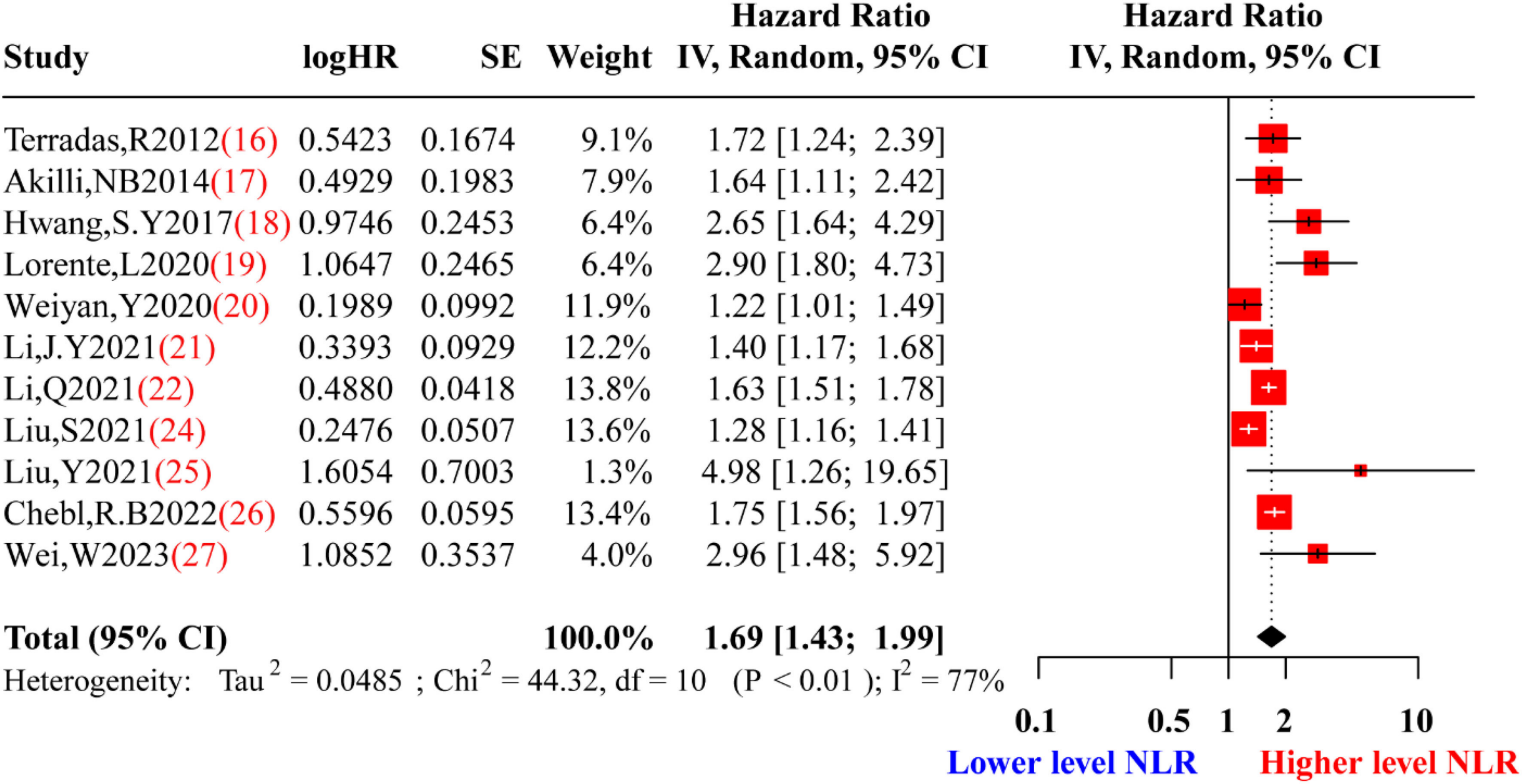
Forest plot of re-synthesis after eliminating one study identified by sensitivity analysis. HR, Hazard Ratio; SE, Standard Error; NLR, neutrophil-to-lymphocyte ratio. Red squares represent the point estimates of the HR of each study, with 95% CI indicated by horizontal bars. Black diamond represent the summary estimate from the pooled studies with 95% CI.

After eliminating highly sensitive studies, a re-synthesis of the included studies showed that a higher level of NLR remained associated with a poorer prognosis for adult sepsis patients (HR: 1.6884, 95% CI: 1.4338-1.9882) with moderate heterogeneity (I^2 =^ 77%, p<0.0001), using a random-effects model (**Figure 4**). And in order to investigate the correlation of the NLR to severity of adult sepsis of this study, we also made a dose-response analysis of this meta-analysis. The dose-response curve from **Supplementary Figure 1** indicated that despite the relationship between the HR of sepsis and the value of NLR was not the linear, nevertheless, in general, the increase in NLR value significantly increases the hazard ratio of mortality in sepsis patients. As for the predictive value of NLR, we made a pooled receiver operating characteristic(ROC) curve, from **Supplementary Figure 2**, we can find sensitivity and specificity of NLR for predicting mortality of adult sepsis were 0.64(0.54-0.74) and 0.79(0.74-0.84) respectively, and the pooled area under curve(AUC) was 0.80(0.76-0.83) which indicated a moderate predictive capability.

### 3.6 Subgroup analysis and meta-regression

**Figure 5:**
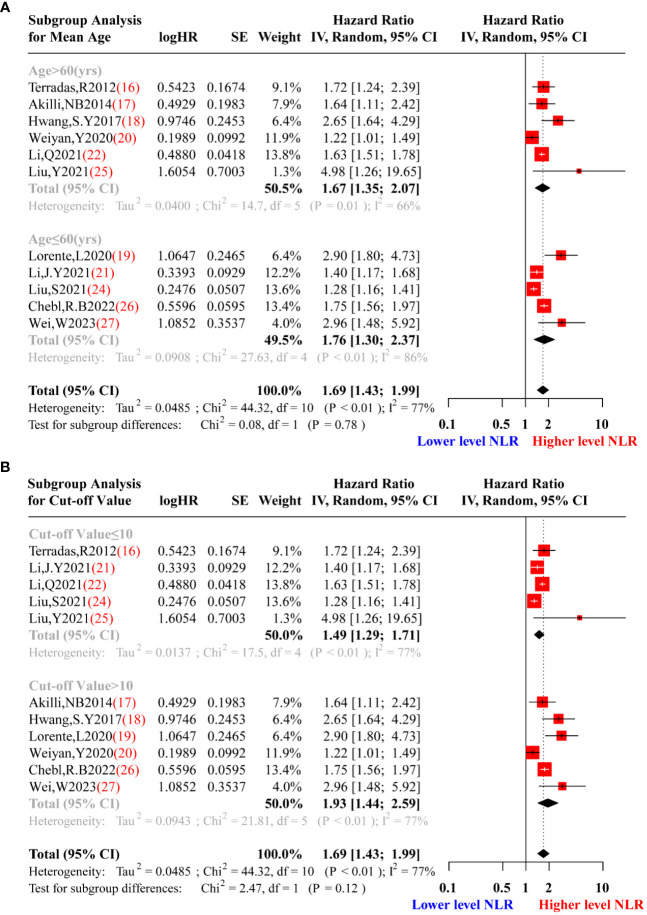
Subgroup analysis sepsis patients’ of mean age **(A)** and cut-off value of the NLR **(B)**. **(A)**.Red squares represent the point estimates of the HR of each study according to mean age subgroup grouping, with 95% CI indicated by horizontal bars. Upper black diamond showed the summary estimate from the pooled studies with >60 years old population demographic characteristics, the middle black diamond showed the pooled studies with ≤60 years old, and the lower black diamond indicated the total summary estimate of the HR of all studies.**(B)**. Red squares represent the point estimates of the HR of each study according to cut-off value of the NLR subgroup grouping, with 95% CI indicated by horizontal bars. Upper black diamond showed the summary estimate from the pooled studies with cut-off value of the NLR ≤10, middle black diamond showed the pooled studies with cut-off value of the NLR >10, and the lower black diamond indicated the total summary estimate of the HR of all studies.

**Figure 6:**
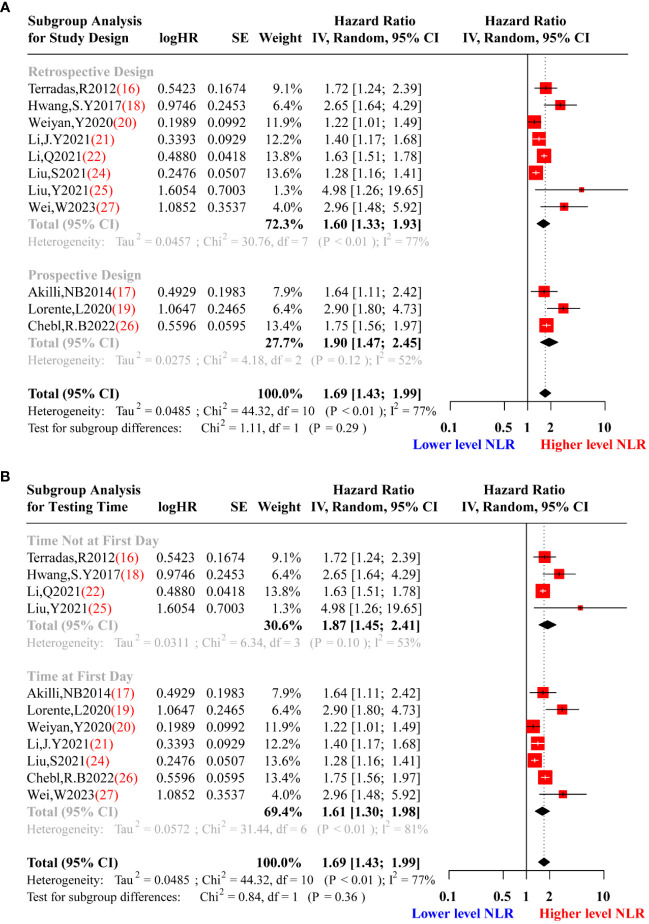
Subgroup analysis of study design **(A)** and testing time of NLR **(B)**. **(A)**.Red squares represent the point estimates of the HR of each study according to study design subgroup grouping, with 95% CI indicated by horizontal bars. Upper black diamond showed the summary estimate of the retrospective studies, the middle black diamond showed the pooled studies of prospective studies, and the lower black diamond indicated the total summary estimate of the HR of all studies. **(B)**. Red squares represent the point estimates of the HR of each study according to testing time of NLR subgroup grouping, with 95% CI indicated by horizontal bars. Upper black diamond showed the summary estimate of the studies with testing time of NLR not on the first day of hospitalization, the middle black diamond showed the pooled studies with testing time of NLR on the first day of hospitalization, and the lower black diamond indicated the total summary estimate of the HR of all studies.

In the process of meta-analysis, adjusting for confounding factors was important because it provided interpretability for the heterogeneity of results. Given that moderate heterogeneity was observed in the data re-synthesis using the random-effects model, we performed subgroup analysis and meta-regression to adjust for confounding factors and explore potential sources of heterogeneity. Stratification was conducted based on the confounding factors named average age of patients (>60 or ≤60 years), study design (prospective or retrospective), cut-off NLR value (>10 or ≤10), and the testing time of NLR (on the first day or not on the first day during hospitalization). **Figure 5A, B** showed the results of the subgroup analysis for mean age and cut-off value, indicating significant heterogeneity in both the subgroup tests and the total test (P<0.05). Nevertheless, for the subgroup analysis of study design, there was a significant difference in heterogeneity between prospective (Tau^2 =^ 0.0275, I^2 =^ 52%, P=0.12) and retrospective designs (Tau^2 =^ 0.0457, I^2 =^ 77%, P<0.01) **Figure 6A**. Furthermore, during subgroup analysis according to NLR testing time, NLR testing on the first day was associated with higher heterogeneity (Tau^2 =^ 0.0572, I^2 =^ 81%, P<0.01), while testing not on the first day showed lower heterogeneity (Tau^2 =^ 0.0311, I^2 =^ 53%, P=0.10) **Figure 6B**, suggesting that study design and NLR testing time were the main sources of heterogeneity in this meta-analysis. To validate the results of the subgroup analysis, we performed meta-regression, and the results presented in **Table 2** corroborated our earlier findings. Through the meta-regression analysis, we incorporated the variables of study design and NLR testing time, which revealed a Tau^2^ of heterogeneity as 0.0012. This value was 0.0473 lower than the Tau^2^ obtained from the previous data synthesis (Tau^2 =^ 0.0485, **Figure 3**). Consequently, it can be inferred that the study design and NLR testing time accounted for approximately 97.53% of the heterogeneity observed in this meta-analysis, consistent with the findings from the subgroup analysis.

**Table 2:** Meta-regression analysis of study design and NLR testing time in adult sepsis patients.

	Tau^2^	z-value	p-value	HR	Lower 95%CI	Upper 95%CI
**Intercept**	0.0012	9.2776	<0.0001	0.5855	0.4618	0.7092
**Study design**		-3.9957	<0.0001	-0.3130	-0.4666	-0.1595
**NLR testing time**		3.5874	0.0003	0.2464	0.1118	0.3810

## 4 Discussion

Sepsis is a severe life-threatening condition associated with an infection characterized by multiple mechanisms, including cytokines, cell death, and dynamic expression of cellular biomarkers, which can lead to circulatory abnormalities and multiple organ failure (28, 29). It is now understood that during the early stages of sepsis, the neutrophil and lymphocyte count rapidly increase due to microbial infection stimulation. However, with disease progression, neutrophils migrate to the infection site, while lymphocytes decrease due to immune suppression, providing the rationale for the individual changes in neutrophil or lymphocyte counts and highlighting their limited predictive value for sepsis prognosis (30, 31).

The NLR reflects the balance between neutrophil and lymphocyte levels and has become a readily available biomarker for adult sepsis in clinical practice. It offers convenience in measurement, low technical requirements, and cost-effectiveness (32, 33). An increasing body of evidence from recently published studies indicates the importance of NLR in predicting the prognosis of adult sepsis (34–36). However, a recent study by Schupp et al. (14) raised concerns, suggesting that NLR might not be a reliable parameter to differentiate between patients with sepsis and septic shock and predict 30-day survival. This discrepancy in findings has led to the lack of consensus over the predictive value of NLR for adult sepsis prognosis. While a meta-analysis (37) has already investigated the prognostic value of NLR in adult sepsis, its results may be biased due to the data extraction method. Some included studies (38, 39) lacked hazard ratios but provided odds ratios as effect indicators for data synthesis. However, it should be borne in mind that OR does not consider the time factor experienced at the endpoint, which results in the loss of important information compared to HR.

**Figure 7:**
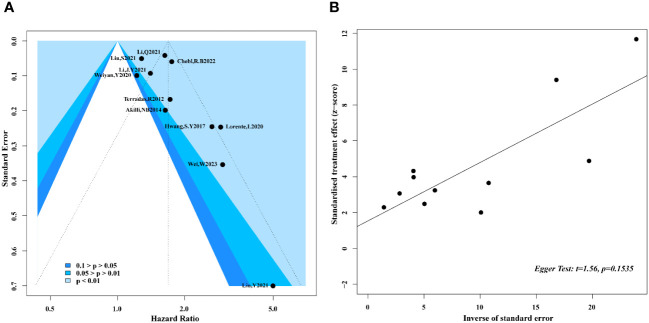
Enhanced contour funnel plot and Egger test for publication bias. **(A)** Enhanced contour funnel plot indicated that all of the studies fell into the blue region, which mean that funnel plots was basically symmetrical; **(B)** Egger test with a result of p=0.1535 illustrated that the publication bias of this meta-analysis was not obvious.

In our meta-analysis, we conducted a rigorous screening process and only included studies that provided hazard ratios and their corresponding 95% confidence intervals. In cases where HR was not directly provided, we utilized Kaplan-Meier curves to indirectly extract the HR using Engauge Digitizer software. A total of 12 studies, comprising 10,811 adult sepsis patients, were included in our analysis. The preliminary synthesis revealed a significant association between higher NLR levels and poor prognosis in adult sepsis patients. To investigate the source of this heterogeneity, we performed a sensitivity analysis, which identified one study (23) as a potential source of high sensitivity. After omitting this study, the heterogeneity was reduced by approximately 10% with the random-effects model. The re-synthesized results after sensitivity analysis did not substantially alter the preliminary synthesis, but the heterogeneity was greatly reduced. To further explore the heterogeneity, we conducted subgroup analysis and meta-regression. The results from both methods indicated that study design and NLR testing time were potential contributors to the observed heterogeneity. Specifically, the type of study design (prospective or retrospective) and the timing of NLR testing during hospitalization appeared to influence the variability in the results. We also examined the possibility of publication bias using enhanced contour funnel plots and Egger’s test. The funnel plot exhibited a symmetric distribution of the included studies, and Egger’s test suggested that there was no significant publication bias in our meta-analysis (p > 0.05) (**Figure 7**).

The clinical value of NLR on the prognosis of septicemia patients is mainly reflected in the following aspects. Firstly, NLR can be used as an important biomarker of the severity sepsis assessing, higher NLR values usually indicate that the patient has a severe infection and inflammatory response (39), from this meta-analysis, the dose-reaction analysis also indicated increase in NLR value significantly increases the hazard ratio of mortality in sepsis patients. Secondly, higher NLR values are often associated with poor prognosis, such as death and complications (14, 19). Therefore, by monitoring changes in NLR values, deterioration can be detected in time and appropriate therapeutic measures can be taken. Meanwhile, according to the change of NLR value, we can make a corresponding treatment program which adjusted to ensure the therapeutic effect.

Nevertheless, our meta-analysis has some limitations. Despite removing one highly sensitive study and conducting a re-synthesis, we still observed moderate heterogeneity even with a random-effects model, potentially affecting the robustness of our conclusions. Secondly, one including study e.g. Terradas et al. (16) defined the disease as bacteremia instead of sepsis, despite a former meta-analysis from Zhiwei Huang (37) included it in their analysis similarly, and we had confirmed that it was not responsible for the heterogeneity of our meta-analysis by sensitive analysis. Thirdly, meta-analysis is prone to be influenced by publication bias, this implied that according to the bias of researchers, that cohort studies with hazard ration around 1 are less likely to be published. Thus, these results should be interpreted with caution due to this selection bias. Additionally, in actual clinical practice, NLR is a biomarker of repeated measurements, the poor prognosis of sepsis can be predicted by the high level of NLR, and the appropriate treatment can affect subsequent level of NLR. Therefore, NLR plays the role of confounder in the former situation and the role of mediator in the latter situation, traditional methods for controlling confounding variables are no longer applicable. Based on the above principles, Zhang, Z. et al (40) recommended structural modeling with inverse probability weighting (IPW) to infer causality from observational data, which played an important decision-making role in clinical management. The link between NLR and mortality of sepsis may be affected by many factors and their causal relationship is largely unknown, further studies using advanced statistical approaches to reveal causality between NLR and adult sepsis are looking forward to investigating.

## 5 Conclusion

NLR is a valuable biomarker for predicting the prognosis of adult sepsis, as higher NLR levels indicate poorer outcomes in adult patients with sepsis. However, more research is needed to understand the relationship between NLR and sepsis prognosis. Large-scale, multiple-center, and high-quality randomized controlled trials with long follow-up periods are warranted to validate the findings of this meta-analysis in the future.

## Data availability statement

The raw data supporting the conclusions of this article will be made available by the authors, without undue reservation.

## Author contributions

HW: Conceptualization, Formal Analysis, Writing – original draft, Writing – review & editing. TC: Methodology, Resources, Writing – review & editing. TJ: Writing – review & editing. YL: Resources, Writing – review & editing. JH: Writing – review & editing. KM: Funding acquisition, Supervision, Writing – original draft.
