# Supplementary material for: Predictive value of the neutrophil-to-lymphocyte ratio in the prognosis and risk of death for adult sepsis patients: a meta-analysis
Source: Front Immunol. 2024 Mar 18;15:1336456. doi: 10.3389/fimmu.2024.1336456 (PMC10982325; doi:10.3389/fimmu.2024.1336456)
Supplement: Supplementary file 1 [file DataSheet_1.pdf]

Supplementary Materials of “Predictive value of the neutrophil-to-lymphocyte ratio in the prognosis and risk of death for adult sepsis patients: a meta-analysis”

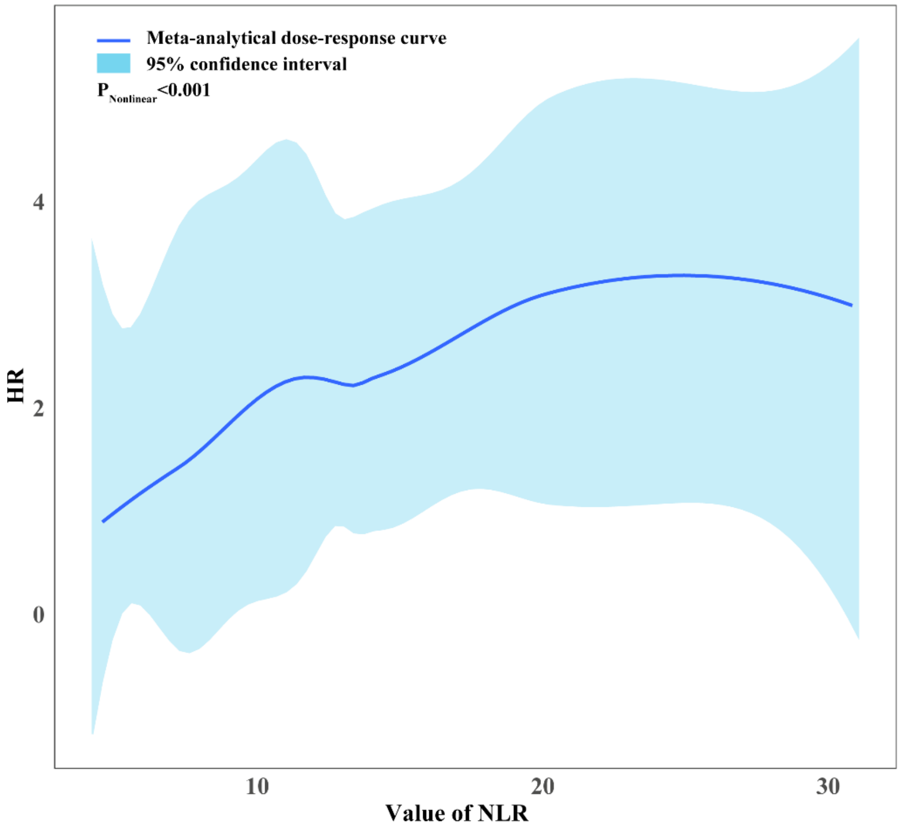

Supplementary Fig1: Fit curve and its corresponding 95%CI of dose-response analysis between the value of NLR and hazard ratio(HR) of adult sepsis mortality.

The x-axis represented the value of NLT, while the y-axis represented the hazard ratio of adult sepsis mortality. Dark blue fit line indicated dose-response curve which did not conform to a linear regression relationship with $P_{Nonlinear}<0.001$, light blue shadow represented the corresponding 95%CI of the fit curve.

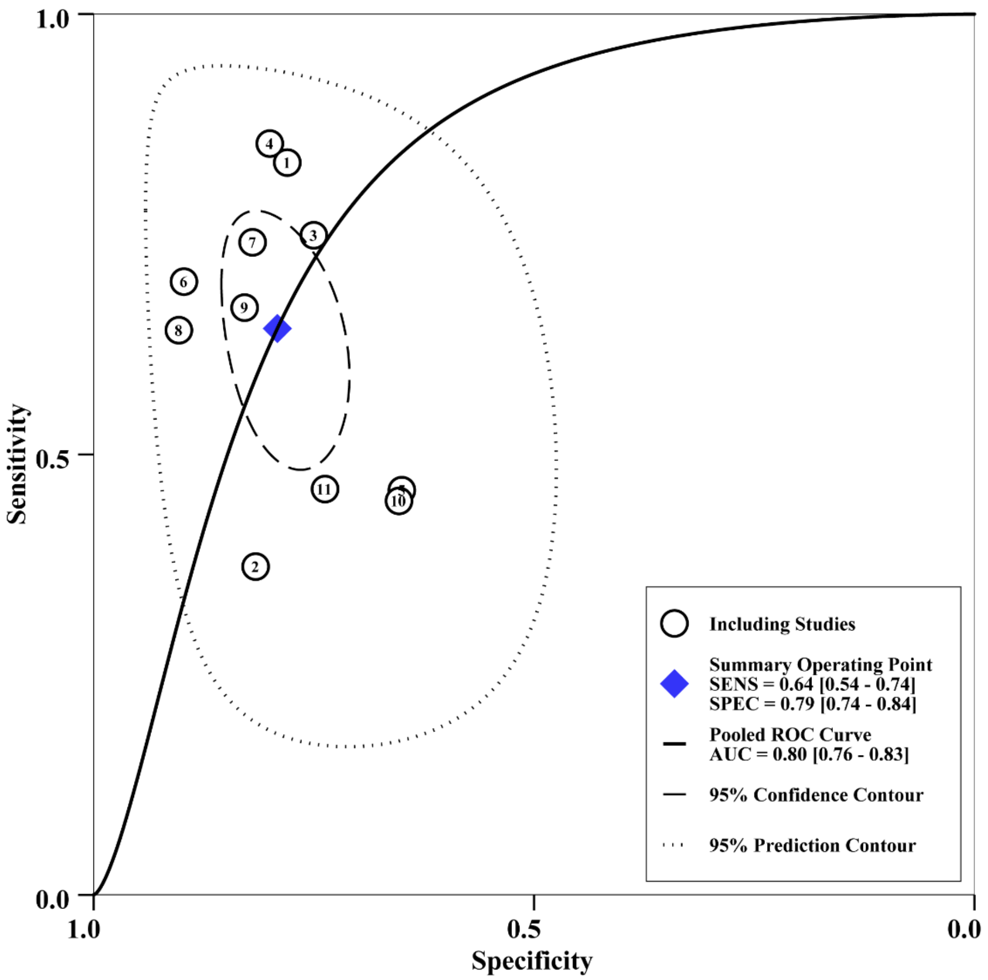

Supplementary Fig2: The pooled ROC and AUC of NLR for predicting mortality of adult sepsis.

ROC= Receiver Operating Characteristic, AUC=Area Under Curve, SENS=Sensitivity, SPEC=Specificity.

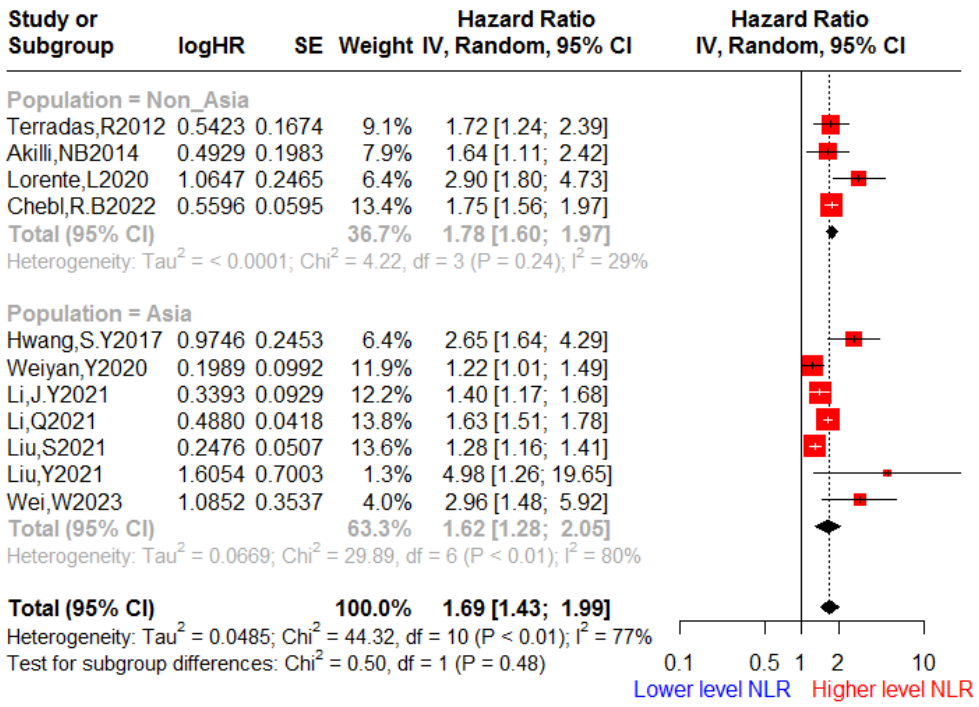

Supplementary Fig3: Subgroup analysis of population. Non-Asia population with heterogeneity test I^2=29%, which indicated minor heterogeneity, while Asia population with heterogeneity test I2=80%, which showed significant heterogeneity.

Supplementary Table1: Continues and categorical variables of age, NLR cut-off and sampling day

Author	HR	Low_CI	Up_CI	Patients NO.	Categorical Age	Continues Age	Categorical Time	Continues Time	Categorical Cut-off	Continues Cut-off
Terradas,R	1.720	1.240	2.390	2311	>60	67.7	Not first day	2	≤10	7.0
Akilli,NB	1.637	1.110	2.415	373	>60	74	First day	1	>10	11.9
Hwang,S.Y	2.650	1.640	4.290	1608	>60	65	Not first day	2	>10	31.0
Lorente,L	2.900	1.800	4.730	203	≤60	60	First day	1	>10	12.1
Weiyan,Y	1.220	1.010	1.490	3043	>60	67	First day	1	>10	20.3
Li,J.Y	1.404	1.170	1.684	274	≤60	57.68	First day	1	≤10	5.5
Li,Q	1.629	1.509	1.778	1245	>60	69.56	Not first day	3	≤10	6.6
Liu,S	1.281	1.159	1.414	264	≤60	52.92	First day	1	≤10	4.9
Liu,Y	4.980	1.262	19.647	91	>60	65	Not first day	7	≤10	8.5
Chebl,R.B	1.750	1.560	1.970	874	≤60	53	First day	1	>10	14.2
Wei,W	2.960	1.480	5.920	309	≤60	57.8	First day	1	>10	13.2

Supplementary Table2: Meta-regression base on the categorical variables of age, sampling day and NLR cut-off value.

Categorical variables	Estimate	SE	z-value	p-value	Low 95%CI	Upper 95%CI
Age(>60, or ≤60)	0.3907	0.1442	2.7085	0.0068	0.1080	0.6734
Sampling day(First day, or not)	0.6354	0.1722	3.6894	0.0002	0.2978	0.9729
NLR cut-off value(≤10 or >10)	-0.3919	0.1125	-3.4823	0.0005	-0.6125	-0.1713

Supplementary Table3: Meta-regression base on the continues variables of age, sampling day and NLR cut-off value.

Continues variables	Estimate	SE	z-value	p-value	Low 95%CI	Upper 95%CI
Age	-0.0108	0.0142	-0.7626	0.4457	-0.0387	0.0170
Sampling day	0.5468	0.1654	2.9866	0.0081	0.2131	0.8357
NLR cut-off value	0.0176	0.0129	1.3591	0.1741	-0.0078	0.0430
